# A PK/PD study comparing twice-daily to once-daily dosing regimens of ertugliflozin in healthy subjects

**DOI:** 10.5414/CP203343

**Published:** 2019-02-25

**Authors:** Vikas Kumar Dawra, Yali Liang, Haihong Shi, Almasa Bass, Anne Hickman, Steven G. Terra, Susan Zhou, David Cutler, Vaishali Sahasrabudhe

**Affiliations:** 1 Pfizer Inc., Groton, CT,; 2 Pfizer Inc., Durham, NC,; 3 Pfizer Inc., Andover, MA, and; 4 Merck & Co., Inc., Kenilworth, NJ, USA; *At the time of study conduct

**Keywords:** ertugliflozin, metformin, type 2 diabetes mellitus, pharmacokinetics, pharmacodynamics

## Abstract

Objective: Ertugliflozin is approved in the US and European Union as a stand-alone product for adults with type 2 diabetes mellitus as once daily (QD) dosing. The approved fixed-dose combination (FDC) of ertugliflozin and immediate-release metformin is dosed twice daily (BID). This study assessed steady-state pharmacokinetics (PK; area under the concentration-time curve over 24 hours (AUC_24_)) and pharmacodynamics (PD; urinary glucose excretion over 24 hours (UGE_24_)) for ertugliflozin 5 and 15 mg total daily doses administered BID or QD. Materials and methods: In this open-label, two-cohort, randomized, multiple-dose, crossover study, healthy subjects received ertugliflozin 2.5 mg BID and 5 mg QD (n = 28) or ertugliflozin 7.5 mg BID and 15 mg QD (n = 22) for 6 days. Plasma and urine samples were collected for 24 hour post morning dose on day 6 in each period. Results: The geometric mean ratio (GMR) (90% CI) of ertugliflozin AUC_24_ was 100.8% (98.8%, 102.8%) for 2.5 mg BID vs. 5 mg QD, and 99.7% (97.1%, 102.5%) for 7.5 mg BID vs. 15 mg QD. GMR (90% CI) of UGE_24_ for BID vs. QD administration was 110.2% (103.0%, 117.9%) at a total daily dose of 5 mg, and 102.8% (97.7%, 108.1%) at 15 mg. The 90% CIs of the GMR of AUC_24_ and UGE_24_ for BID vs. QD dosing were within the acceptance range for equivalence (80 – 125%) and the prespecified criterion for similarity (70 – 143%), respectively. All treatments were well tolerated. Conclusion: There are no clinically meaningful differences in steady-state PK or PD between ertugliflozin BID and QD regimens at total daily doses of 5 and 15 mg, supporting BID administration of ertugliflozin as a component of the ertugliflozin/metformin (immediate-release) FDC.

## Introduction 

The global prevalence of type 2 diabetes mellitus (T2DM) is increasing and is projected to reach more than 623 million people by 2045; this is largely due to the aging population and rising obesity rates [1]. In the US, T2DM represents a large medical burden, with an estimated 9.4% of the population having the disease and ~ 7.2 million adults remaining undiagnosed [2]. 

Ertugliflozin is a selective inhibitor of sodium-glucose cotransporter 2 (SGLT2) [3, 4] that is approved by the US Food and Drug Administration and the European Medicines Agency in the EU for the treatment of adults with T2DM [5]. SGLT2 is primarily located in the proximal tubule of the kidney and is responsible for the reabsorption of ~ 90% of glucose from the urine [6]. SGLT2 inhibitors reduce renal glucose reabsorption and lower renal threshold for glucose excretion, thereby increasing urinary glucose excretion (UGE). This leads to a reduction in plasma glucose levels and glycated hemoglobin (A1C) in patients with T2DM. Phase III studies have shown that ertugliflozin, when dosed once daily (QD) at 5 mg or 15 mg, significantly reduces A1C, body weight, and blood pressure [7, 8, 9, 10, 11]. 

Ertugliflozin is a Biopharmaceutics Classification System Class 1 drug owing to its high permeability and high solubility [12]. Studies have shown that the pharmacokinetics (PK) of ertugliflozin are similar in healthy subjects and patients with T2DM [13], and the oral absorption of ertugliflozin is rapid, with median time to maximum plasma concentration (t_max_) occurring at ~ 1 hour post dose in the fasted state and ~ 2 hours post dose in the fed state [14]. Food does not have a clinically meaningful effect on the exposure of ertugliflozin; it can therefore be administered without regard to meals [14]. Absolute bioavailability of ertugliflozin is ~ 100% [15]. Ertugliflozin exposure increases in a dose-proportional manner over the dose range 0.5 – 300 mg [3]. 

The mean elimination half-life for ertugliflozin is estimated to be 15.2 hours for healthy volunteers, and 16.6 hours for patients with T2DM and normal renal function [13]. Consistent with the ertugliflozin half-life and linear PK, the mean day 14 : day 1 area under the plasma concentration-time curve (AUC) accumulation ratio ranges from 1.2 to 1.4, and steady-state concentrations are achieved 4 – 6 days after initiating QD dosing. Glucuronidation is the major metabolic pathway with minor contributions from oxidative metabolism. Renal excretion of unchanged ertugliflozin accounts for 1.5% of administered dose [4]. The primary uridine diphosphate glucuronosyltransferase (UGT) involved in the glucuronidation of ertugliflozin is UGT1A9, with additional contribution from UGT2B7 [3]. 

Phase I single and multiple ascending dose studies of ertugliflozin in healthy subjects have demonstrated that cumulative UGE over a period of 24 hours (UGE_24_) increases in a dose-related manner [16]. UGE_24_ values are generally similar on day 1 and at steady state for the respective ertugliflozin dose [16]. Compared with healthy subjects, ertugliflozin 15 mg induces higher median UGE_24_ in patients with T2DM and normal renal function, as expected with higher circulating glucose levels in patients with T2DM [17]. Dose-response modeling indicated that ertugliflozin 5 mg and 15 mg result in near maximal UGE_24_ and A1C lowering (glycemic efficacy), with the 15-mg dose providing incrementally greater UGE and A1C lowering relative to the 5-mg dose [7, 8, 9]. 

Metformin is a biguanide antihyperglycemic agent that decreases hepatic glucose production and increases insulin-mediated glucose uptake, thus improving glycemic control [18, 19, 20]. A fixed-dose combination (FDC) of ertugliflozin and metformin is also approved in the US and EU for the treatment of adults with T2DM [21]. The FDC formulation contains an immediate release formulation of metformin, which is dosed twice daily (BID); as such, the ertugliflozin/metformin FDC is also recommended to be dosed BID. This is in contrast to the QD dosing of ertugliflozin evaluated in phase III studies. Therefore, this study was designed to assess whether ertugliflozin exposure as assessed by AUC over 24 hours (AUC_24_) at steady state is equivalent, and whether UGE_24_ at steady state is similar, at the same total daily dose, irrespective of whether ertugliflozin is dosed QD or BID. 

## Materials and methods 

### Study objectives 

The primary objectives of this phase I, open-label, two-cohort, two-period, crossover study were to demonstrate the equivalence of ertugliflozin exposure (AUC_24_) on day 6 (steady state) and the similarity of UGE_24_ at total daily doses of 5 mg or 15 mg when administered QD vs. BID (2.5 mg BID vs. 5 mg QD, and 7.5 mg BID vs. 15 mg QD) in healthy subjects. The QD and BID treatments would be considered equivalent with respect to exposure at steady state if the 90% confidence interval (CI) for the geometric mean ratio (GMR) for AUC_24_ of the BID and QD dosing regimens fell within 80 – 125%. The equivalence bounds were chosen in line with the FDA guidance for demonstrating bioequivalence [22]. The QD and BID treatments would be considered similar with respect to UGE_24_ at steady state if the 90% CI for the GMR of UGE_24_ of the BID and QD dosing regimens fell within 70 – 143%. In phase III studies for oral antidiabetic agents, the non-inferiority bound for difference in baseline-corrected A1C is generally set at ≤ 0.3%. Ertugliflozin at 5 mg and 15 mg resulted in placebo-adjusted least-squares mean A1C reductions from baseline of 0.7% and 0.9%, respectively, on a metformin background [9]. The non-inferiority bound of 0.3% for A1C therefore represents preservation of ~ 0.4% of the effect on A1C, or ~ 60% of the overall treatment effect at the 5-mg ertugliflozin dose. Therefore, the similarity bounds proposed in this current study correspond to the preservation of 70% of the total effect to establish non-inferiority of the PD endpoint between the two dosing regimens, assuming one-to-one translation of A1C to UGE_24_. 

The secondary objective was to evaluate the safety and tolerability of ertugliflozin at total daily doses of 5 mg or 15 mg when administered QD vs. BID for 6 days in healthy subjects. 

### Participants 

Healthy male and female subjects aged 18 – 55 years with body mass index 17.5 – 30.5 kg/m^2^ and total body weight > 50 kg, who had provided informed consent and were willing and able to comply with the study plan, were eligible for inclusion in the study. Exclusion criteria included: evidence of glucosuria by urine dipstick test at screening or day 0 (period 1); screening fasting plasma glucose ≥ 100 mg/dL or A1C ≥ 5.7%; estimated glomerular filtration rate < 90 mL/min/1.73m^2^ based on the 4-variable Modification of Diet in Renal Disease equation [23]; positive urine screen for drugs of abuse or recreational drugs; history of alcohol abuse or binge drinking, and/or any other illicit drug use or dependence within 6 months of screening; evidence of a clinically significant malabsorption condition; known hypersensitivity or intolerance to any SGLT2 inhibitor or any component of the formulations; pregnant or breastfeeding women. All subjects who had taken prescription or nonprescription drugs (except hormonal methods of birth control and post-menopausal treatment), vitamins and dietary supplements within 7 days or 5 half-lives (whichever was longer) prior to first dose of ertugliflozin were also excluded from the study. 

### Study design and treatment 

The study was conducted in compliance with the ethical principles of the Declaration of Helsinki and the all International Conference on Harmonisation Good Clinical Practice guidelines. 

The study consisted of a screening visit and two treatment periods; screening occurred within 28 days of the first dose of study medication in period 1. In this two-cohort study, eligible subjects were randomized to one of the two treatment sequences to receive ertugliflozin 2.5 mg BID and 5 mg QD or ertugliflozin 7.5 mg BID and 15 mg QD for 6 days. Subjects who received the QD dose in period 1 received the BID dose in period 2 and vice versa, with dosing in consecutive crossover periods separated by a washout period of ≥ 7 days. Subjects were admitted to the clinical research unit on day 0 of each period and received the assigned treatment in the morning (QD dosing) or morning and evening (BID dosing) on days 1 – 6. The morning dose was administered after an overnight fast of ≥ 10 hours, and the evening dose was administered ~ 12 hours after the morning dose and 1 hour before dinner. Identical, standardized meals and snacks were provided on days 5 and 6 of each period; each subject had to completely consume all food (including snacks) on day 6 of each period. Each subject voided their bladder just prior to the morning dose on day 6. Subjects were also asked to void as close as possible to the end of each prescribed urine interval (0 – 6, 6 – 12, 12 – 18, and 18 – 24 hours). 

The original protocol included two cohorts: cohort A (n = 20) received 2.5 mg BID and 5 mg QD, and cohort B (n = 28) received 7.5 mg BID and 15 mg QD. However, in 16 subjects in cohort A, the 24-hour urine collection following the morning dose on day 6 was not performed as per protocol. This systemic error in urine collection in cohort A resulted in incomplete urine collection over the 24-hour period post morning dose on day 6, which could have significantly impacted UGE_24_, the primary pharmacodynamic (PD) endpoint of the study. Therefore, the protocol was amended to add one replacement cohort (cohort C: 2.5 mg BID and 5 mg QD; n = 22). All subjects in cohorts B and C were analyzed for PK and PD parameters. All subjects in cohorts A, B, and C were analyzed for safety (Supplemental Table 1). 

### Assessments 

**Pharmacokinetics and pharmacodynamics**

For each period, blood samples for PK analysis were collected for QD dosing as follows: on days 4, 5, and 6 before administration of the morning dose, and at 0.5, 1, 2, 3, 4, 8, 12, and 24 hours after the morning dose on day 6. For BID dosing, blood samples were collected at 0.5, 1, 2, 3, 4, 8, 12 (pre-evening dose), 12.5, 13, 14, 15, 16, 20, and 24 hours after the morning dose on day 6. 

Plasma samples were analyzed for ertugliflozin concentrations using a validated high-performance liquid chromatography tandem mass spectrometric method, as described previously [24]. The calibration curve was linear over the range 0.500 – 500 ng/mL. 

For each period, urine for analysis of glucose was collected for 24 hours on day 6, in four 6-hour intervals: 0 – 6, 6 – 12, 12 – 18, and 18 – 24 hours. Urine samples were analyzed for glucose concentrations using a coupled enzymatic assay (Cobas 6000 Glucose Hexokinase Assay) with a lower limit of quantification (LLOQ) of 2.00 mg/dL. Calibration standard responses were linear over the range of 2.00 – 717 mg/dL, using a two-point calibration. Those samples with concentrations above the upper limit of quantification were adequately diluted into the calibration range. The between-day assay accuracy, expressed as percent relative error (%RE), for quality control (QC) concentrations ranged from –9.0 to –6.7% for the low, medium, high, and dilution QC samples. Assay precision, expressed as the between-day percent coefficient of variation (%CV) of the mean estimated concentrations of QC samples was ≤ 1.0% for the low (54.9 mg/dL), medium (127 mg/dL), high (528 mg/dL), and dilution (622 mg/dL at 4 × dilution or 2,488 mg/dL back-calculated) QC samples. 

**Safety**

All subjects who received at least 1 dose of study medication were included in the safety analysis. Safety assessments, including physical examination, monitoring of adverse events (AEs), blood pressure, pulse rate, and measurement of clinical laboratory parameters, were conducted from screening and throughout the duration of study participation. Subjects received a follow-up phone call 14 ± 3 days after administration of the last dose of study medication in period 2, to assess for AEs. AEs were coded using the Medical Dictionary for Regulatory Activities (MedDRA version 18.1). 

### Statistical analysis 

A sample size of 18 completed subjects per cohort provided 99% and 97% power, respectively, so that equivalence for AUC_24_ and similarity for UGE_24_ could be demonstrated. Subjects with significant protocol deviations or observations that could impact the PK and/or UGE_24_ (e.g., incomplete/missing urine interval collection, failure to completely consume all food on day 6 in each period, vomiting post meals, incorrect/missing dose) were to be replaced. The statistical analysis for UGE_24_ included data from all subjects who consumed 100% of the meals provided (breakfast, lunch, dinner, and snacks) on day 6 in at least one period. 

PK parameters were calculated for each subject and for each treatment using non-compartmental analysis of plasma concentration-time data. Samples below the LLOQ were set to 0 for analysis. PD parameters (UGE_24_ and percent inhibition of glucose reabsorption) were also calculated for each subject. UGE_24_ was calculated as cumulative urinary glucose excreted over a period of 24 hours. Inhibition of glucose reabsorption was expressed as a ratio of 24-hour urinary glucose excretion to the product of estimated glomerular filtration rate and mean plasma glucose over 24 hours. Natural log-transformed AUC_24_ and UGE_24_ on day 6 for QD and BID ertugliflozin were analyzed for each cohort separately using a mixed-effects model, with sequence, period, and treatment as fixed effects, and subject within sequence as a random effect. 

The adjusted mean difference and 90% CI were exponentiated to provide estimates of the GMR (Test : Reference (BID : QD)) and 90% CI for the ratio. The BID and QD dosing regimens were considered equivalent for AUC_24_ and similar for UGE_24_ if the 90% CI for the GMR was within the range 80 – 125% and 70 – 143%, respectively. 

## Results 

### Subject demographics 

70 subjects were enrolled: 20 in cohort A (2.5 mg BID and 5 mg QD), 28 in cohort B (7.5 mg BID and 15 mg QD), and 22 in cohort C (2.5 mg BID and 5 mg QD). All subjects in cohorts A, B, and C were analyzed for safety, and all subjects in cohorts B and C were analyzed for PK and PD. PD data from cohort A were not evaluable due to the significant protocol deviations resulting in incomplete urine collection; therefore, PK parameters and glucose amount in the urine were not determined for cohort A. Demographic characteristics of the study population are outlined in Table 1. The majority of subjects were male and black. Mean age was 34.4 (range 20 – 53) years, and mean (standard deviation) body mass index and body weight were 25.9 (2.6) kg/m^2^ and 78.3 (11.0) kg, respectively. One subject in cohort B was discontinued due to a protocol violation (positive urine drug screen) after receiving ertugliflozin 15 mg QD in period 1. Two subjects in cohort C discontinued from the study (1 due to an AE). These discontinuations were not related to study medication (Supplemental Table 1). 

### Pharmacokinetics 

**Figure 1.**
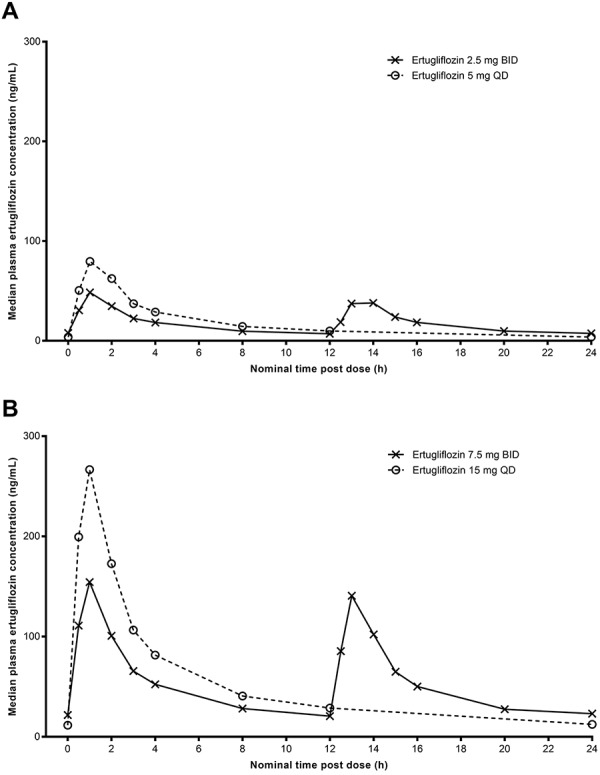
Median plasma ertugliflozin concentration-time profiles on day 6 after multiple QD or BID oral doses. A: Ertugliflozin 2.5 mg BID/5 mg QD. B: Ertugliflozin 7.5 mg BID/15 mg QD. BID = twice daily; QD = once daily.

Median plasma ertugliflozin concentration-time profiles on day 6 following QD or BID dosing are shown in Figure 1. Ertugliflozin PK parameter values are summarized descriptively in Table 2. The geometric mean AUC_24_ was similar for both BID and QD treatments following administration of ertugliflozin 2.5 mg BID vs. 5 mg QD, and 7.5 mg BID vs. 15 mg QD for 6 days. As expected, for both cohorts, the geometric mean maximum observed plasma concentration (C_max_) after the morning dose was higher for the QD than the BID treatment. In addition, it was noted that the geometric mean C_max_ for the BID treatment after the morning dose was slightly higher than the evening dose. Median t_max_ following the morning dose was 1.0 hour for both the QD and the BID treatments in both cohorts. 

The geometric %CV across treatments ranged from 18 to 22% for ertugliflozin AUC_24_ and from 20 to 29% for ertugliflozin C_max_. Steady state was reached by day 4 for all treatments, based on similar median trough (pre-dose) concentrations on days 4, 5, 6, and 7. 

The GMRs (90% CI) of ertugliflozin AUC_24_ for comparisons of 2.5 mg BID vs. 5 mg QD and 7.5 mg BID vs. 15 mg QD are shown in Table 3. The GMRs (90% CI) of ertugliflozin AUC_24_ were 100.8% (98.8%, 102.8%) for 2.5 mg BID vs. 5 mg QD, and 99.7% (97.1%, 102.5%) for 7.5 mg BID vs. 15 mg QD. The 90% CIs of the GMRs for AUC_24_ were within the acceptance range for equivalence (80 – 125%). 

### Pharmacodynamics 

**Figure 2.**
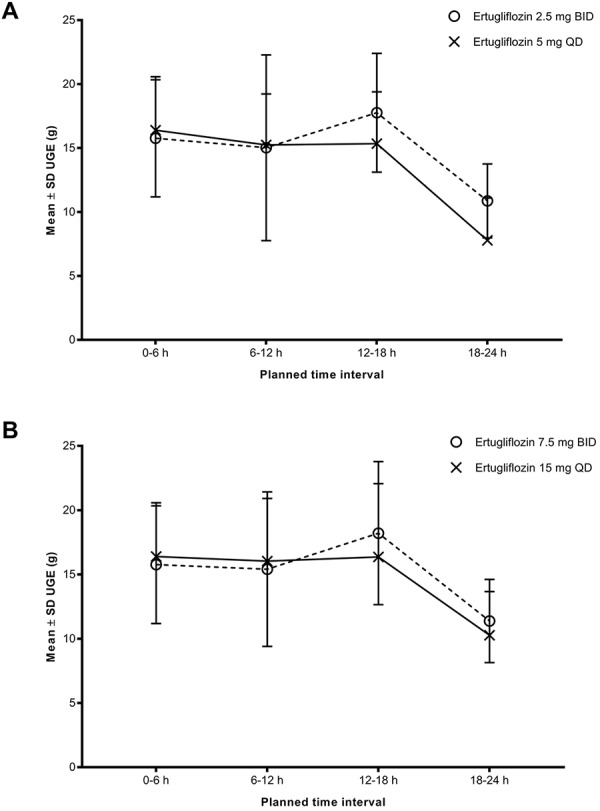
Mean ± SD UGE vs. time intervals for (A) ertugliflozin 2.5 mg BID/5 mg QD and (B) ertugliflozin 7.5 mg BID/15 mg QD. BID = twice daily; QD = once daily; SD = standard deviation; UGE = urinary glucose excretion.

Mean UGE over the intervals 0 – 6, 6 – 12, 12 – 18, and 18 – 24 hours after the morning dose on day 6 (Figure 2) and mean UGE_24_ on day 6 (Table 4) were similar between QD and BID treatments for both cohorts. The GMRs (90% CI) of ertugliflozin UGE_24_ for comparisons of 2.5 mg BID vs. 5 mg QD, and 7.5 mg BID vs. 15 mg QD are shown in Table 3. The GMRs (90% CI) of ertugliflozin UGE_24_ were 110.2% (103.0%, 117.9%) for 2.5 mg BID vs. 5 mg QD, and 102.8% (97.7%, 108.1%) for 7.5 mg BID vs. 15 mg QD. The 90% CIs of the GMRs for UGE_24_ fell within the acceptance range for similarity (70 – 143%) and were also within the acceptance range for equivalence (80 – 125%). The percent inhibition of glucose reabsorption was similar for all treatments, with mean values ranging from 38.5 to 42.0% (Table 4). 

### Safety 

No deaths, or serious or severe AEs occurred. One subject in cohort C was discontinued due to an AE of increased alanine aminotransferase, which was considered by the investigator to be not related to study medication. The most frequently reported treatment-emergent AEs were nausea (10 all-causality events; 7 considered treatment related) and headache (9 all-causality events; all considered treatment related). The majority of treatment-emergent AEs were mild in intensity, with 2 AEs of moderate severity reported by subjects in cohort B (nausea and vomiting) and 5 AEs of moderate severity reported in cohort C (nausea, arthropod bite, vomiting, vulvovaginal mycotic infection, and genitourinary tract infection), all of which resolved. 

## Discussion 

Ertugliflozin and its two fixed-dose combinations (ertugliflozin/sitagliptin and ertugliflozin/metformin) were recently approved in the US and the EU for the treatment of adults with T2DM. FDC formulations have been shown to have additional benefits above those provided by combining therapies through co-administration of the individual components. Patients with T2DM who received an FDC treatment have demonstrated increased treatment adherence compared with those receiving the same agents as co-administered therapy [25]. FDC formulations have also been associated with reduced T2DM-related and monthly all-cause costs as compared to respective co-administered therapies [26]. 

Ertugliflozin, a selective SGLT2 inhibitor, at doses of 5 mg or 15 mg QD has been shown to produce statistically significant and clinically relevant reductions in A1C, body weight, and blood pressure [7, 8, 9, 10, 11]. In phase III studies, ertugliflozin QD at doses of 5 mg and 15 mg was administered with metformin dosed BID. The ertugliflozin/metformin FDC contains the immediate-release formulation of metformin, which is recommended to be administered BID; as such, the ertugliflozin/metformin FDC is also recommended to be dosed BID. In order to bridge the BID dosing regimen of ertugliflozin as a component of the ertugliflozin/metformin FDC with the QD dosing used in the phase III clinical development program, this phase I study was designed to demonstrate the equivalence of ertugliflozin AUC_24_ and the similarity of ertugliflozin UGE_24_ when administered QD vs. BID in healthy subjects at steady state, using the phase III ertugliflozin doses (5 mg and 15 mg). 

Several phase I studies have been conducted to assess the clinical pharmacology of ertugliflozin in healthy volunteers and in patients with T2DM. PK of ertugliflozin was predictable and similar across different study populations (i.e., healthy, obese, T2DM) [13]. In addition, based on a population PK analysis of ertugliflozin, age did not have a clinically meaningful effect on ertugliflozin PK [13]. As per the dose vs. A1C response analysis of ertugliflozin, no dosage adjustment is recommended on the basis of age. Also, no clinically meaningful effect on the PK of ertugliflozin was observed when administered with food, and the dose-response relationship for UGE was similar under fasted and fed conditions [14, 16]. Therefore, the results of the current study, conducted under fasted conditions in younger, healthy subjects, are applicable to ertugliflozin administered under fed conditions, which is the dosing recommendation for ertugliflozin/metformin FDC for a typical T2DM patient. 

One of the primary objectives of the present study was to demonstrate the equivalence of steady-state ertugliflozin exposure (AUC_24_) on day 6 of treatment at total daily dosing of 5 mg and 15 mg when administered QD vs. BID in healthy subjects (5 mg QD and 2.5 mg BID; 15 mg QD and 7.5 mg BID). The 90% CIs of the GMRs for AUC_24_ fell within the acceptance range for equivalence (80 – 125%). As expected, the observed C_max_ after an ertugliflozin QD dosing regimen was higher than that for BID dosing. These study results demonstrate equivalent exposure of ertugliflozin after BID compared with QD administration at the same total daily dose, thus supporting the BID administration of ertugliflozin in the ertugliflozin/metformin FDC. 

In a previous phase I study, ertugliflozin PK and UGE were assessed after single day dosing of 2 mg and 4 mg administered as a single dose (given at breakfast) and split into two doses (given at breakfast and lunch) in patients with T2DM. Plasma samples for PK evaluation and urine samples for UGE were collected up to 24 hours after administration of the morning dose. At each dose level, total ertugliflozin plasma exposure observed with single and split doses was similar, as was the UGE_24_. In addition, the dose-response relationship for steady-state UGE was explored for patients with T2DM and healthy volunteers [27]. The results suggest that the dose-response relationship for steady-state UGE between patients with T2DM and healthy subjects are similar, except that dose to half-maximal response (ED_50_) in healthy subjects is significantly higher than that in patients with T2DM. Because of the higher ED_50_ for ertugliflozin in healthy subjects, there is higher sensitivity to test similarity of UGE in healthy subjects than in patients with T2DM, as the UGE response in healthy subjects at the doses evaluated in the current study is further removed from the maximal response. Therefore, the current study was designed in healthy subjects to evaluate the similarity of steady-state UGE_24_ when ertugliflozin is dosed as QD or BID at the same total daily dose. 

The QD and BID treatments were to be considered similar with respect to steady-state UGE_24_ if the 90% CI for the GMR of UGE_24_ of the BID and QD dosing regimens fell within the prespecified range of 70 – 143%. The PD results from this study indicate that the UGE_24_ at steady state was similar when administered as BID vs. QD (2.5 mg BID vs. 5 mg QD, and 7.5 mg BID vs. 15 mg QD), as the 90% CI for the GMR of UGE_24_ of BID and QD dosing regimens fell within this prespecified range of similarity. In addition, while not prespecified, the GMR of UGE_24_ of BID and QD dosing regimens also fell within the acceptance range of equivalence (80 – 125%). Furthermore, the mean UGE over the intervals of 0 – 6, 6 – 12, 12 – 18, and 18 – 24 hours were comparable between the QD and BID treatments. These PD results strongly support that there are no meaningful differences in the PD (UGE_24_) profile of ertugliflozin between BID and QD regimens in healthy subjects. 

Taken together, the findings of this study indicate that there are no meaningful differences in the PK or PD properties of ertugliflozin when administered either as BID or QD at total daily doses of 5 mg and 15 mg. In addition, both dosing regimens were well tolerated in healthy subjects. The results also indicate that the efficacy of ertugliflozin, when administered BID as a component of the ertugliflozin/metformin FDC, can be expected to be similar to that observed when ertugliflozin was administered QD in phase III trials, and support the use of ertugliflozin dosed BID with metformin in the FDC formulation. 

## Conclusion 

There are no clinically meaningful differences in the PK or PD between ertugliflozin BID and QD regimens at total daily doses of 5 mg and 15 mg. Ertugliflozin can be administered BID as a component of the ertugliflozin/metformin FDC formulation. 

## Data sharing statement 

Upon request, and subject to certain criteria, conditions, and exceptions (see https://www.pfizer.com/science/clinical-trials/trial-data-and-results for more information), Pfizer will provide access to individual de-identified participant data from Pfizer-sponsored global interventional clinical studies conducted for medicines, vaccines, and medical devices (1) for indications that have been approved in the US and/or EU or (2) in programs that have been terminated (i.e., development for all indications has been discontinued). Pfizer will also consider requests for the protocol, data dictionary, and statistical analysis plan. Data may be requested from Pfizer trials 24 months after study completion. The de-identified participant data will be made available to researchers whose proposals meet the research criteria and other conditions, and for which an exception does not apply, via a secure portal. To gain access, data requestors must enter into a data access agreement with Pfizer. 

## Funding 

This study was funded by Merck Sharp & Dohme Corp., a subsidiary of Merck & Co., Inc., Kenilworth, NJ, USA (MSD), in collaboration with Pfizer Inc., New York, NY, USA. Statistical support was provided by Lata Maganti of MSD and Kyle Matschke of Pfizer Inc. Medical writing support was provided by Beth Elam, PhD, of Engage Scientific Solutions (Horsham, UK) and was funded by Pfizer Inc. and MSD. 

## Conflict of interest 

HS, AH, SGT, and VS are employees of Pfizer Inc. VKD, YL, and AB were employees of Pfizer Inc. at the time of study conduct. SZ is an employee of Merck Sharp & Dohme Corp., a subsidiary of Merck & Co., Inc., Kenilworth, NJ, USA (MSD). DC was an employee of MSD at the time of study conduct. 

**Table 1.** Baseline characteristics.

	Total daily dose 5 mg (n = 22)	Total daily dose 15 mg (n = 28)
Sex, n		
Male	12	21
Female	10	7
Age, years		
Mean (SD)	34.8 (8.0)	32.4 (7.0)
Range	22 – 53	20 – 45
Race, n		
White	6	3
Black	11	20
Asian	3	0
Other	2	5
Ethnicity, n		
Hispanic/Latino	5	6
Non-Hispanic/Latino	17	22
Weight, kg		
Mean (SD)	77.2 (14.1)	79.6 (8.4)
Range	59.4 – 108.9	55.9 – 97.1
BMI, kg/m^2^		
Mean (SD)	26.2 (2.9)	26.1 (2.3)
Range	20.7 – 30.2	21.9 – 30.3

BMI = body mass index; n = number of subjects; SD = standard deviation.

**Table 2.** Descriptive summary of plasma ertugliflozin PK parameter values on day 6.

	PK parameter summary statistics by treatment			
	Total daily dose 5 mg		Total daily dose 15 mg	
	Ertugliflozin 2.5 mg BID	Ertugliflozin 5 mg QD	Ertugliflozin 7.5 mg BID	Ertugliflozin 15 mg QD
N, n	22, 20	22, 22	27, 26	28, 28
AUC_24_, ng×h/mL	399.2 (18)	397.9 (18)	1,192 (20)	1,193 (22)
C_max1_, ng/mL	47.5 (25)^a^	81.3 (29)	154.2 (20)	268.2 (20)
t_max1_, h	1.0 (0.5 – 1.1)^a^	1.0 (0.5 – 2.1)	1.0 (0.5 – 2.0)	1.0 (0.5 – 2.1)
C_max2_, ng/mL	42.8 (28)	NA	140.1 (21)	NA
t_max2_, h	2.0 (1.0–2.1)	NA	1.0 (1.0–2.0)	NA

Values are geometric mean (geometric percent coefficient of variation) for AUC_24_ and C_max_; or median (range) for t_max_. C_max1_ and t_max1_ indicate post morning dosing for the BID regimen. C_max2_ and t_max2_ indicate post evening dosing for the BID regimen. AUC_24_ = area under the plasma concentration-time curve over 24 hours; BID = twice daily; C_max_ = maximum observed plasma concentration; N = number of subjects in the treatment group; n = number of subjects contributing to the summary statistics; NA = not assessed; PK = pharmacokinetics; QD = once daily; t_max_ = time to maximum plasma concentration. ^a^21 subjects were included in summary statistics for C_max1_ and t_max1_.

**Table 3.** Statistical summary of treatment comparisons for plasma ertugliflozin AUC24 and UGE24 on day 6.

Parameter (unit)	Comparison (test vs. reference)	Adjusted (least-squares) geometric means		Ratio (BID:QD) of adjusted means	90% CI for ratio
		BID (Test)	QD (Reference)		
AUC_24_, ng×h/mL	Ertugliflozin 2.5 mg BID vs. 5 mg QD	401.0	397.9	100.8	98.8, 102.8
	Ertugliflozin 7.5 mg BID vs. 15 mg QD	1,190.0	1,193.0	99.7	97.1, 102.5
UGE_24_, g	Ertugliflozin 2.5 mg BID vs. 5 mg QD	57.0	51.7	110.2	103.0, 117.9
	Ertugliflozin 7.5 mg BID vs. 15 mg QD	58.8	57.3	102.8	97.7, 108.1

Adjusted geometric means were obtained using a mixed-effects model (separate for each cohort) with sequence, period, and treatment as fixed effects and subject within sequence as a random effect. The adjusted mean difference and 90% CI were exponentiated to provide estimates of the geometric mean ratio (Test : Reference (BID : QD)) and 90% CI for the ratio. Ratios (and 90% CIs) are expressed as percentages. AUC_24_ = area under the plasma concentration-time curve over 24 hours; BID = twice daily; CI = confidence interval; QD = once daily; UGE_24_ = urinary glucose excretion over 24 hours.

**Table 4.** Descriptive summary of UGE24 and inhibition of glucose reabsorption.

Parameter (units)		Total daily dose 5 mg		Total daily dose 15 mg	
		Ertugliflozin 2.5 mg BID	Ertugliflozin 5 mg QD	Ertugliflozin 7.5 mg BID	Ertugliflozin 15 mg QD
UGE_24_, g	n	20	20	21	23
	Geometric mean	57.1	52.5	58.6	57.6
	%CV	31	34	28	28
Inhibition of glucose reabsorption, %	n	20	20	21	23
	Arithmetic mean	40.3	38.5	42.0	40.6
	%CV	27	29	26	26

Values are geometric mean and geometric %CV for UGE_24_; or arithmetic mean and arithmetic %CV for inhibition of glucose reabsorption. BID = twice daily; %CV = percent coefficient of variation; n = number of subjects contributing to the summary statistics; QD = once daily; UGE_24_ = urinary glucose excretion over 24 hours.

## Supplemental material

Supplemental Table 1. Subject disposition.
