# Supplementary material for: A PK/PD study comparing twice-daily to once-daily dosing regimens of ertugliflozin in healthy subjects
Source: Int J Clin Pharmacol Ther. 2019 Feb 25;57(4):207–16. doi: 10.5414/CP203343 (PMC6528385; doi:10.5414/CP203343)
Supplement: Supplemental Table 1. [file intjclinpharmacol-57-207-S01.pdf]

Supplemental Table 1. Subject disposition.

| Number of subjects | Cohort A Total daily dose 5 mg | | Cohort B Total daily dose 15 mg | | Cohort C Total daily dose 5 mg | |
|---|---|---|---|---|---|---|
| | **Ertugliflozin 2.5 mg BID** | **Ertugliflozin 5 mg QD** | **Ertugliflozin 7.5 mg BID** | **Ertugliflozin 15 mg QD** | **Ertugliflozin 2.5 mg BID** | **Ertugliflozin 5 mg QD** |
| **Assigned to study medication** | 70 | | | | | |
| Treated | 20 | 20 | 27 | 28 | 22 | 22 |
| Completed | 20 | 20 | 27 | 27 | 20 | 22 |
| Discontinued | 0 | 0 | 0 | 1 | 2 | 0 |
| Protocol violation | 0 | 0 | 0 | 1 | 0 | 0 |
| No longer willing to participate in study | 0 | 0 | 0 | 0 | 1 | 0 |
| Adverse events | 0 | 0 | 0 | 0 | 1 | 0 |
| **Analyzed for PK** | | | | | | |
| PK concentration | 20 | 20 | 27 | 28 | 21[a] | 22 |
| PK parameter | 0 | 0 | 27 | 28 | 21[a] | 22 |
| **Analyzed for PD** | | | | | | |
| PD | 0 | 0 | 27 | 28 | 21[a] | 22 |
| **Analyzed for safety** | | | | | | |
| Adverse events | 20 | 20 | 27 | 28 | 22 | 22 |
| Laboratory data | 20 | 20 | 27 | 28 | 22 | 22 |

BID = twice daily; PD = pharmacodynamics; PK = pharmacokinetics; QD = once daily.

Discontinuations were attributed to the last study medication received.

[a]One subject discontinued on day 2 in period 2 (ertugliflozin 2.5 mg BID), and consequently had no PK data for period 2.
